# Pure shift amide detection in conventional and TROSY-type experiments of ^13^C,^15^N-labeled proteins

**DOI:** 10.1007/s10858-022-00406-z

**Published:** 2022-11-18

**Authors:** Jens D. Haller, Andrea Bodor, Burkhard Luy

**Affiliations:** 1 Institute of Organic Chemistry and Institute for Biological Interfaces 4 – Magnetic Resonance, Karlsruhe Institute of Technology (KIT), Hermann-Von-Helmholtz-Platz 1, 76344 Eggenstein-Leopoldshafen, Germany; 2 Institute of Chemistry, Analytical and BioNMR Laboratory, ELTE –Eötvös Loránd University, Pázmány Péter Sétány 1/A, 1117 Budapest, Hungary

**Keywords:** High resolution, Homonuclear decoupling, Pure shift, Solution state, IDPs, Amide detection, Proteins, BIRD, Real-time

## Abstract

Large coupling networks in uniformly ^13^C,^15^N-labeled biomolecules induce broad multiplets that even in flexible proteins are frequently not recognized as such. The reason is that given multiplets typically consist of a large number of individual resonances that result in a single broad line, in which individual components are no longer resolved. We here introduce a real-time pure shift acquisition scheme for the detection of amide protons which is based on ^13^C-BIRD^r,X^. As a result the full homo- and heteronuclear coupling network can be suppressed at low power leading to real singlets at substantially improved resolution and uncompromised sensitivity. The method is tested on a small globular and an intrinsically disordered protein (IDP) where the average spectral resolution is increased by a factor of ~ 2 and higher. Equally important, the approach works without saturation of water magnetization for solvent suppression and exchanging amide protons are not affected by saturation transfer.

**Supplementary Information:**

The online version contains supplementary material available at 10.1007/s10858-022-00406-z.

## Introduction

Modern NMR spectroscopy is an indispensable tool for obtaining valuable information at atomic resolution. However, this information may be obliterated if the studied biomolecules contain repeating units, or they belong to the group of intrinsically disordered proteins (IDPs)—where chemical shifts are distributed close to random coil values and very little dispersion is experienced (Dyson and Wright 2001). Large efforts have been made to overcome signal overlap, either by performing experiments at higher fields, by applying detection of heteronuclei (Serber et al. 2000; Bermel et al. 2006, 2008, 2012; Felli and Pierattelli 2014; Chhabra et al. 2018) or by introducing experiments of high dimensionality (Bermel et al. 2009; Narayanan et al. 2010; Bertini et al. 2011). Another possibility is offered by so-called pure shift methods that allow the collapse of the broad, sometimes unresolved multiplets to a single line—which has the advantage to increase both sensitivity and resolution for fast tumbling proteins. However, from the many available pure shift methods (Zangger 2015; Castañar and Parella 2015; Hammarström and Otting 1994; Kupče and Wagner 1995; Castañar et al. 2013; Ying et al. 2014; Kiraly et al. 2015; Im et al. 2019; Haller et al. 2019; Bodor et al. 2020; Meyer and Zangger 2014, 2013; Zangger and Sterk 1997; Nilsson and Morris 2007; Sakhaii et al. 2009; Aguilar et al. 2011; Lupulescu et al. 2012; Paudel et al. 2013; Reinsperger and Luy 2014; Foroozandeh et al. 2014; Sinnaeve et al. 2016) only the ones with essentially uncompromised sensitivity may be applicable to biomolecules (Hammarström and Otting 1994; Kupče and Wagner 1995; Castañar et al. 2013; Ying et al. 2014; Kiraly et al. 2015; Im et al. 2019; Haller et al. 2019; Bodor et al. 2020; Meyer and Zangger 2014).

Since detection of protons provides highest sensitivity, it is well-established in most standard experiments of the field (Clore and Gronenborn 1991). In particular amide protons are widely used for detection, and it is worth to address the special challenges for their *pure shift* acquisition in uniformly ^13^C,^15^N-labeled samples. A drawback of this approach is that amide protons being in exchange with water imply the presence of an enormous solvent signal that has to be suppressed during the NMR experiment. Solvent suppression has turned out to be especially challenging for pure shift methods where pulses are applied during the acquisition and water magnetization so far had to be saturated using a weak radiofrequency field (rf) or pulsed field gradients (Castañar et al. 2013; Ying et al. 2014; Kiraly et al. 2015). As a consequence, amide protons in exchange with the solvent suffer from signal attenuation (Grzesiek and Bax 1993; Mori et al. 1995) which is especially striking for IDPs studied close to neutral pH (Bai et al. 1993).

Furthermore, in uniformly ^13^C,^15^N-labeled biomolecules a large number of homo- *and* heteronuclear couplings are encountered that, even if unresolved, cause signal broadening and highest resolution can only be obtained if *all* present couplings are suppressed. Up to now, the suppression of numerous heteronuclear long-range couplings is achieved by using power-intensive composite pulse decoupling (CPD), sometimes even on both heteronuclei, at high strain for probe and hardware. This poses a significant limit to the acquisition time and hence to the achievable resolution (Ying et al. 2014).

Here, we present an acquisition scheme for the pure shift detection of amide protons in uniformly ^13^C,^15^N-labeled proteins, applicable both to small globular proteins and IDPs, that overcomes the mentioned issues. The method is based on the real-time decoupling approach as originally proposed by Lupulescu et al. (Lupulescu et al. 2012). The central element of the decoupling sequence is a ^13^C-BIRD^r,X^ filter (Garbow et al. 1982; Uhrín et al. 1993) that enables suppression of all small homo- and heteronuclear couplings and avoids the need of ^13^C-CPD (Haller et al. 2019; Bodor et al. 2020). As a result, uncompromised acquisition times are accessible and high resolution is achieved with only ^15^N-decoupling to be considered if applicable. Moreover, water polarization can be stored along the z-axis during acquisition—avoiding the need of saturation of the water resonance and allowing the water spin reservoir to be used for fast pulsing experiments (Schanda 2009).

We here use the proposed pure shift acquisition in simple FHSQC (Mori et al. 1995) and BEST-TROSY (Solyom et al. 2013) measurements, but it should be applicable in any ^1^H,^15^N-HSQC or TROSY-type experiment. Results are shown for the small globular protein ubiquitin, and for the intrinsically disordered protein domain p53TAD^1−60^, where a significant enhancement in resolution can be observed at high sensitivity.

## The pulse sequence

Modern real-time homonuclear decoupling requires a *selective inversion element* that allows to distinguish the detected active spins from their passive coupling partners and can hence act as *J*-refocusing block—a pure shift spectrum can then be acquired in a single shot (Lupulescu et al. 2012). For this purpose, the acquisition is repeatedly interrupted and the FID is recorded in chunks. Each time the acquisition is paused, couplings to *passive* spins are refocused by the selective inversion element while acquired *active* spins are *in total* not affected. If the chunk length is chosen short compared to the inverse multiplet width, passive couplings cannot evolve significantly during acquired chunks and decoupling is thus achieved to a good approximation. For the detection of amide protons, the discrimination between active and passive spins can be obtained e.g., with band-selective pulses (Castañar et al. 2013; Ying et al. 2014) or using a ^15^N-BIRD filter (Kiraly et al. 2015).

**Fig. 1:**
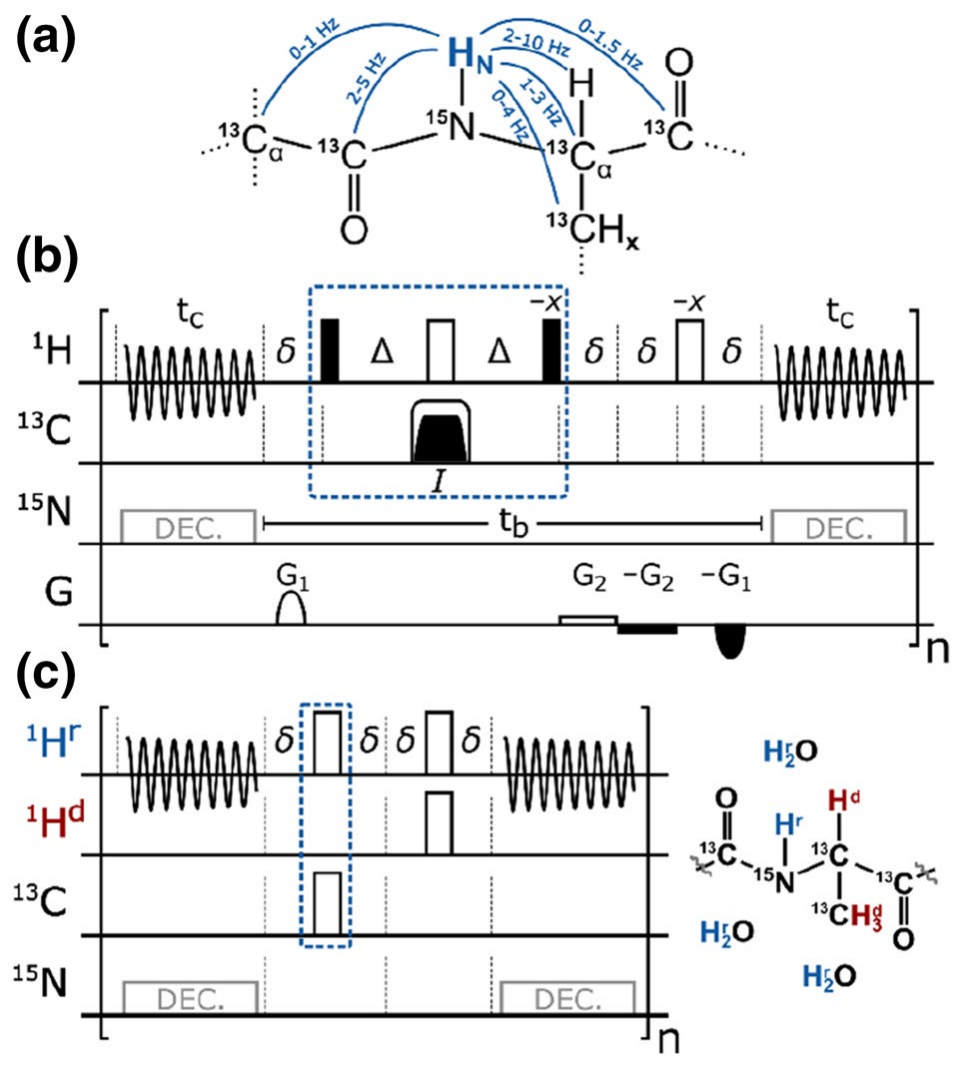
**a** Relevant *J*-couplings for amide protons (Salvador et al. 2011). **b** Pulse sequence for real-time decoupled acquisition of amide protons using a ^13^C-BIRD^*r,X*^ filter (blue dashed box) (Garbow et al. 1982; Uhrín et al. 1993). CPD on ^15^N (in grey) is applied for ^1^H,^15^N HSQC-type experiments, but omitted for TROSY-type experiments. Filled narrow and open wide bars correspond to 90° and 180° pulses, respectively, with phase *x* if not denoted otherwise. A broadband BIP or BIBOP inversion pulse (*I*) (Kobzar et al. 2004, 2008; Ehni and Luy 2013; Smith et al. 2001; Baum et al. 1985) covering ~ 200 ppm is applied with a pulse length of 500 μs. ∆ and δ are set to 1/(2 × ^1^*J*_CαH_) and 0.5 ms, respectively. Gradients are used to remove artifacts (G_1_ ≈ 3.0%) and to avoid radiation damping (G_2_ ≈ 0.1%). In order to achieve acquisition times of > 200 ms, the number of chunks is typically set to n = 8 with half chunk lengths of t_c_ = 14–18 ms and breaks of t_b_ ≈ 9.2 ms. **c** The effective rotations of the BIRD^r,X^ element are illustrated for directly ^13^C-bound (H^d^, red) and remote (H^r^, blue) protons.

For uniformly ^13^C,^15^ N-labeled proteins, we propose the approach shown in Fig. 1. In the given pure shift acquisition sequence (Fig. 1b), the selective inversion element consists of a ^13^C-BIRD^*r,X*^ filter in combination with a hard 180° pulse on the proton channel. Following the nomenclature of Uhrín et al., the BIRD-filter selectively inverts remote protons (*r*) and carbons (*X*), while protons directly bound to ^13^C (*d*) are not inverted (Uhrín et al. 1993). For clarity, the pulse sequence is again shown in Fig. 1c using a pseudo pulse sequence where the BIRD-filter is reduced to the effective pulses of the element for remote (^1^H^*r*^) and directly ^13^C-bound protons (^1^H^*d*^). It is obvious that acquired amide protons (as part of ^1^H^*r*^) experience two inversions and, if relaxation and pulse imperfections are neglected, they are thus not affected by the selective inversion element. On the other hand, all carbons as well as protons directly attached to ^13^C (^1^H^*d*^) experience only one effective inversion. Therefore, all coupling partners of the amide protons are inverted by the *J*-refocusing element (except ^15^N) leading to the real-time suppression of both homonuclear ^1^H^r^,^1^H^d^-couplings as well as heteronuclear long-range ^1^H^r^,^13^C-couplings. Carbon decoupling is hence achieved at minimal additional cost of power and long acquisition times are not restricted by the ^13^C-decoupler. Note that nearly all ^2^*J*- and ^3^*J*-couplings of the amide proton are heteronuclear (Fig. 1a) and in total, the numerous couplings can cause substantial broadening. In ^1^H,^15^N-HSQC-type experiments only the large ^1^*J*_NH_ coupling additionally needs to be suppressed by ^15^N-CPD during detection. In TROSY-type experiments, on the other hand, the ^1^*J*_NH_ coupling is supposed to evolve and, practically, there is no rf-limit imposed on the acquisition time.

Furthermore, it is crucial to note that also water belongs to the group of remote protons (^1^H^*r*^) and, to good extent, it is likewise *not* affected by the selective inversion element which has remarkable consequences in terms of solvent suppression.

## Water suppression

The large excess of H_2_O in biomolecular samples prevents the detection of much smaller protein peaks and the water signal intensity has to be reduced drastically. Saturation of the water bulk magnetization from a weak rf-field causes signal attenuation of exchanging amide protons and should hence be avoided (Mori et al. 1995; Hoult 1976). Note, using pulsed field gradients to dephase the water signal can also lead to saturation of amide protons—typical T_1_ relaxation times of water are in the range of several seconds, which is much longer than conventional recovery delays. In order to avoid saturation transfer to exchanging protons (and consecutively also to surrounding spins via NOE and spin diffusion) it is thus advisable to apply so-called flip-back techniques that store the water bulk magnetization along the non-observable + z axis during acquisition (Jahnke and Kessler 1994; Lippens et al. 1995). The bulk water can thereby be used as a large spin reservoir for exchanging protons in fast pulsing experiments that in favorable cases allow a vast reduction of the recovery delay (t_r_) (Mori et al. 1995; Solyom et al. 2013).

With respect to real-time homonuclear decoupling, one should be aware that already a small perturbation to the strong water resonance could cause enormous artifacts. For this reason, common real-time pure shift sequences make use of pulsed field gradients, which are capable to suppress transverse magnetization that originates from pulse imperfections during the chunked acquisition.

**Fig. 2:**
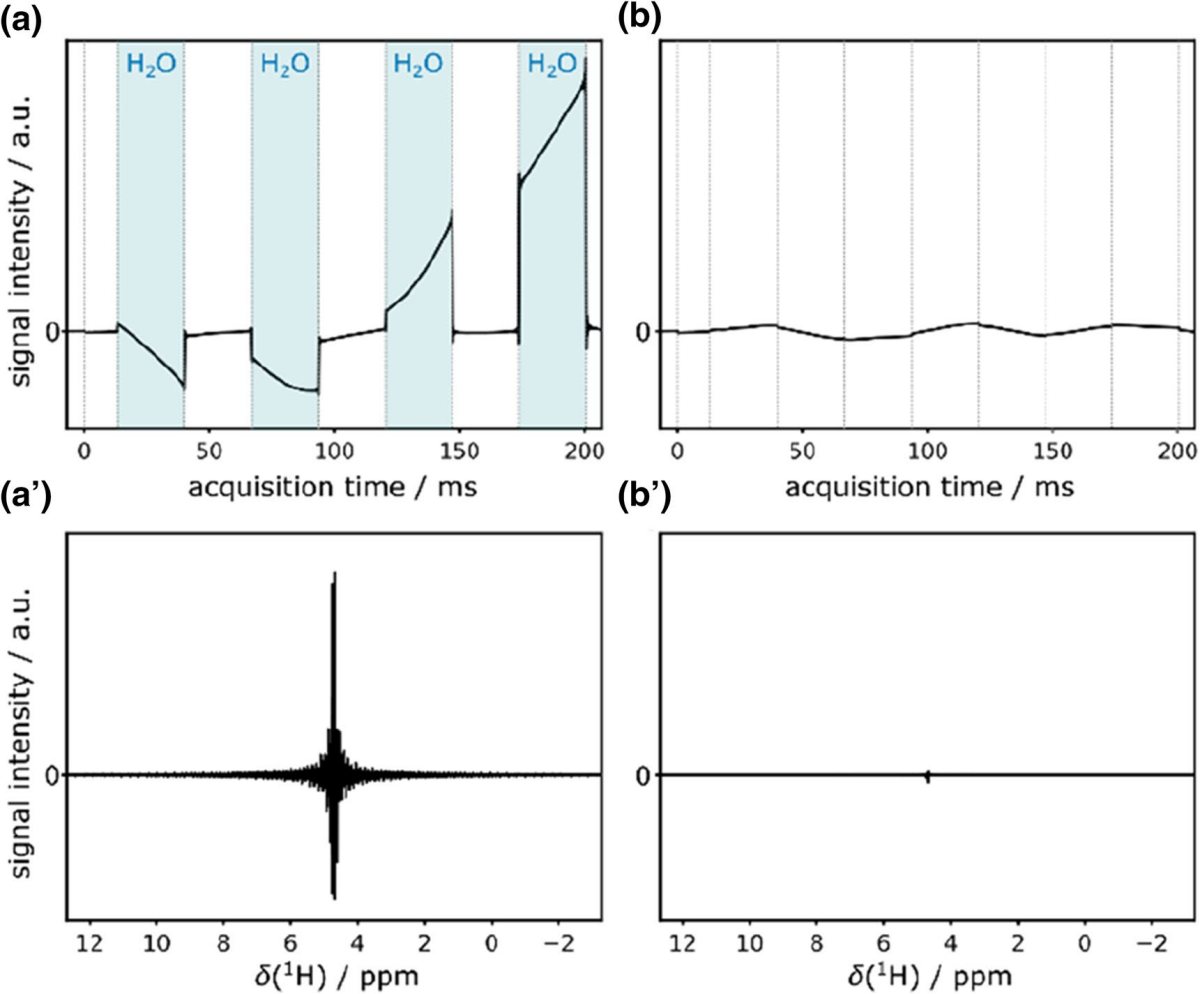
Water artifacts caused by radiation damping in a pure shift FHSQC using a ^15^N-BIRD (left) and ^13^C-BIRD^r,X^ filter (right). Only the first FID was acquired with 16 dummy scans using a cryogenically cooled TCI probehead (**a, b**). Blue areas indicate where water bulk magnetization is flipped to − z axis with strong radiation damping. Respective spectra with resulting artifacts are shown below (**a**ʹ**, b**ʹ)

If, however, the water bulk magnetization has to be flipped by the selective inversion element from the + z to the − z axis, radiation damping will cause artifact buildup *during* detection where pulsed field gradients are not applicable and the strong water signal will reappear. The emerging artifacts in such a case are enormous and are shown for a pure shift FHSQC using ^15^N-BIRD in Fig. 2a and aʹ.

In every second chunk, water is flipped to the − z axis where radiation damping non-linearly rotates the water magnetization and the entire 1D spectrum is dominated by the water chunking artifact. Note that also the use of band-selective homonuclear decoupling would lead to comparable results.

On the other hand, with our proposed sequence using a ^13^C-BIRD^*r,X*^ filter, the water bulk magnetization can be kept along the + z axis and artifacts due to radiation damping can be reduced by two orders of magnitude as shown in Fig. 2b and bʹ. By this means, the quality of water suppression is on an equal level compared to other pure shift methods (see Fig. S1) while the water bulk magnetization can be retained for exchanging amide protons. Bear in mind, that radiation damping strongly depends on the employed probe and the quality of water suppression can vary significantly for different hardware and settings. For best water suppression we recommend to follow the procedure described in the supporting information.

## Materials and methods

NMR spectra were recorded on a 600 MHz Bruker Avance III spectrometer equipped with a 5 mm H-C/N-D TCI cryogenically cooled probehead and a 16.4 T Bruker Avance III spectrometer operating at 700.05 MHz equipped with a 5 mm Prodigy H&F-C/N-D TCI probehead. The composition of the U-^13^C/^15^N labeled protein samples was: 0.5 mM human ubiquitin by Silantes in sealed 5 mm NMR tube, 7% D_2_O, pH 4.7, 30 mM sodium acetate, 50 mM NaCl; 1 mM p53TAD^1−60^, 20 mM MES (2-(*N*-morpholino)ethanesulfonic acid), 20 mM NaCl, 10 mM TCEP (tris (2-carboxyethyl)phosphine), 10% D_2_O, pH 6.0, prepared as described earlier (Dudas et al. 2020).

## Application to proteins

**Fig. 3:**
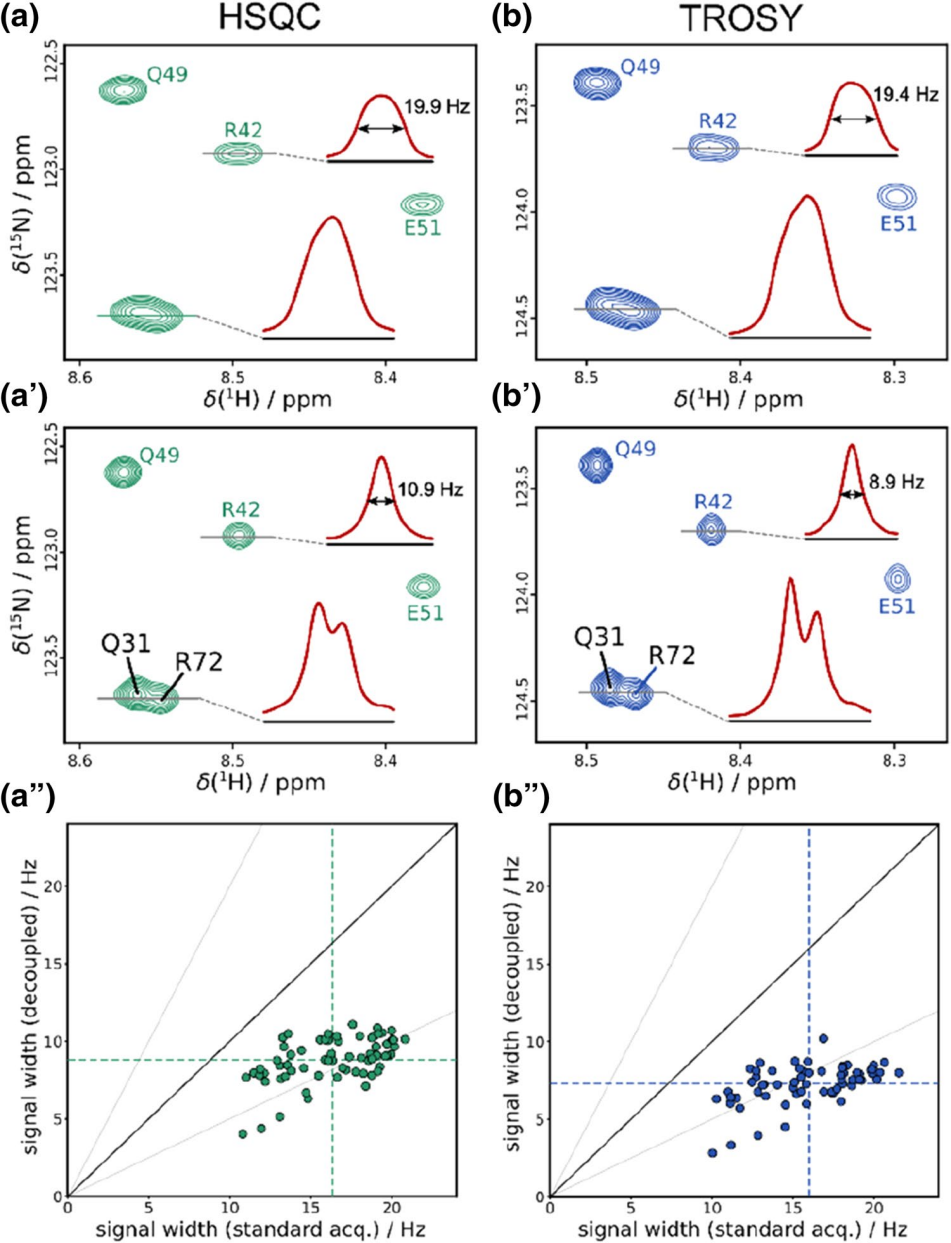
Spectra of ubiquitin acquired at 600 MHz using FHSQC (left) and BEST-TROSY (right) with standard acquisition (**a, b**) and ^13^C-BIRD^r,X^-based homonuclear decoupling (**a**ʹ**, b**ʹ). While in the FHSQC 2048 complex points were acquired in 214 ms with t_c_ = 17.8 ms and n = 6, for TROSY 3096 complex points were recorded in 321 ms with t_c_ = 20 ms and n = 8 using a magnetization recovery delay of t_r_ = 1.0 s. The assignments are based on literature (Weber et al. 1987; Wang et al. 1995) and overlapping signals (Q31 and R72) can be resolved in (**a**ʹ**, b**ʹ). Correlation plots are shown for the FHSQC and BEST-TROSY in (**a**ʹʹ**, b**ʹʹ), respectively, where signal widths with standard acquisition and homonuclear decoupling are compared. Dashed lines mark average values and a quadratic phase-shifted sine (**a, b, a**ʹ**, b**ʹ) and no apodization (**a**ʹʹ**, b**ʹʹ) was used for processing

Due to the sheer number of amide proton couplings in uniformly ^13^C,^15^N-labeled proteins, the splittings contribute significantly to the linewidths of small globular or intrinsically disordered proteins, where natural linewidths are expected to be relatively narrow. In many such cases, unresolved multiplets likely dominate the actual signal width and pure shift methods may lead to an increase in spectral resolution and sensitivity. The first example is given in Fig. 3 for the 76 residue long folded ubiquitin, where homo- and long-range heteronuclear couplings are removed using the ^13^C-BIRD-based pure shift acquisition in an FHSQC and BEST-TROSY. A closer analysis of the highlighted slices through selected cross peaks shows that signals obtained by standard detection exhibit a very broad top of the signal that indicates the presence of multiplets. If these underlying multiplets are collapsed, sharp Lorentzian-type singlets are obtained. As seen in Fig. 3, the standard acquisition scheme results in signal widths of 10–23 Hz for both FHSQC and TROSY spectra, with slightly narrower lines for the TROSY as expected. Introduction of the ^13^C-BIRD^r,X^-based pure shift acquisition (aʹ, bʹ) results in considerable narrowing to approximately half the signal width. The main width reduction is due to the suppression of ^1^H^N^,^1^H-couplings and it should be noted that also long-range ^1^H^N^,^13^C-couplings contribute significantly to line broadening. Suppressing ^1^H^N^,^13^C-couplings in case of ubiquitin we find an average reduction in signal width of 3–4 Hz (Fig. S2). Since decoupling of long-range ^1^H^N^,^13^C-couplings consumes negligible additional power, the achievable resolution is not hardware-limited and for the discussed experiments acquisition times up to 321 ms were used. Moreover, only with full decoupling the favorable relaxation properties of TROSY are revealed. Despite the fact that cross-correlated relaxation is more pronounced for larger proteins, already for ubiquitin we find a considerable increase in resolution using TROSY and overlapping signals as e.g. Q31 and R72 can be resolved unambiguously as highlighted in Fig. 3bʹ.

To provide a wider estimate on the increase in resolution, signal widths at half height for spectra with and without the proposed pure shift sequence were extracted using a semi-automated python script. Correlated values are illustrated in Fig. 3aʹʹ and bʹʹ. In these correlation plots, based on spectra without apodization, we find a reduction in signal width from average values (dashed lines) of 16.3 Hz to 8.8 Hz for the FHSQC and from 16.0 to 7.3 Hz for BEST-TROSY when decoupling is applied. Note that a certain line broadening is induced by quadratic phase-shifted sine apodization and average line widths are increased to 10.7 Hz and 8.3 Hz for the pure shift FHSQC and BEST-TROSY, respectively. Improved linewidths can be obtained using processing parameters as described elsewhere (Kiraly et al. 2018).

**Fig. 4:**
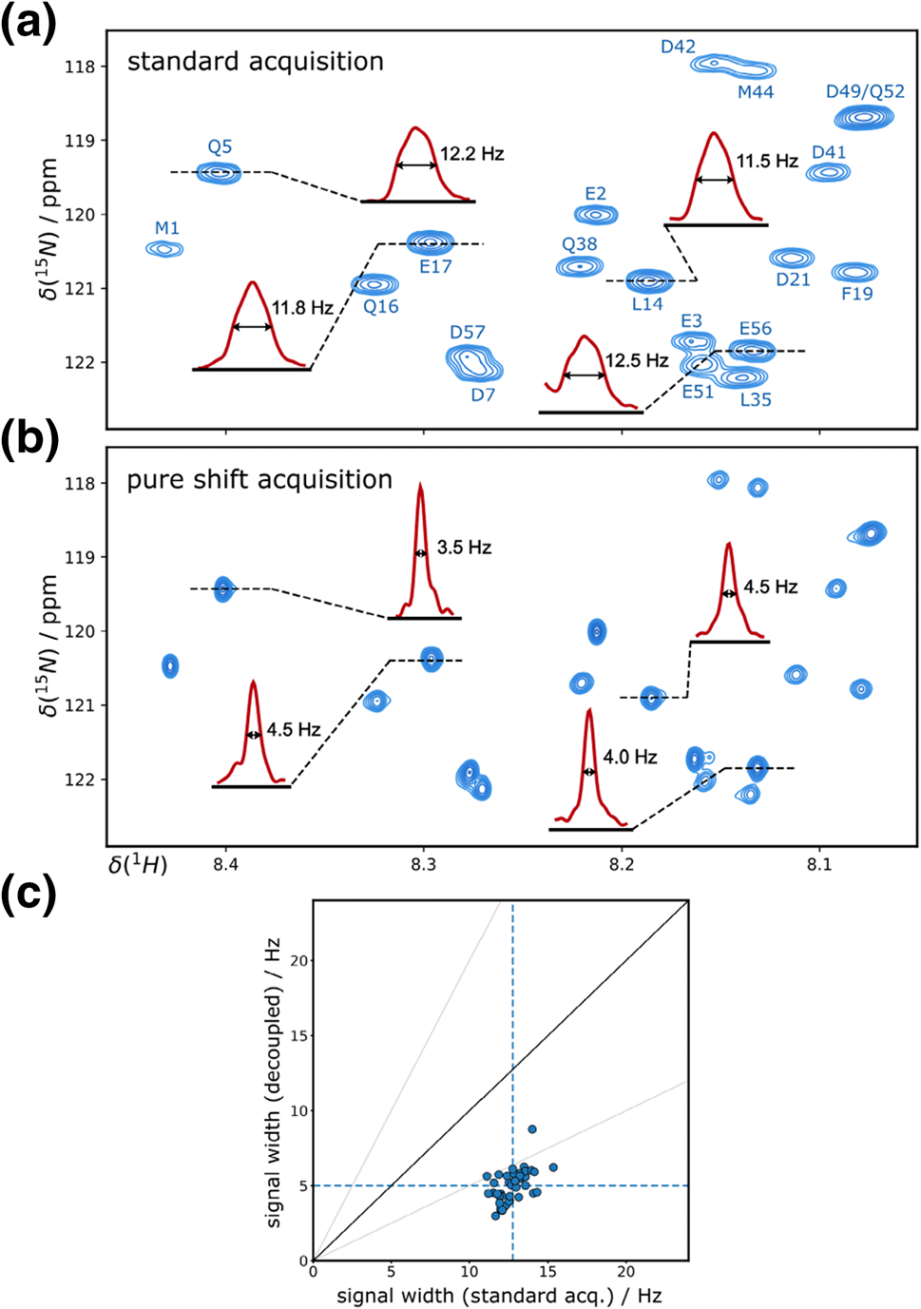
Spectra of p53TAD^1-60^ were acquired at 700 MHz using BEST-TROSY with standard acquisition (**a**) and homonuclear decoupling (**b**). 2048 complex points were acquired in 243 ms with t_c_ = 15.2 ms and n = 8 at a temperature of 298 K and a recovery delay of t_r_ = 200 ms. Signal widths are compared using correlation plots (**c**) where dashed lines mark average values and no apodization was used for processing in (**a–c**)

Based on the fact that transverse relaxation is typically much slower for intrinsically disordered proteins, in this case even sharper lines are expected using the ^13^C-BIRD^r,X^ pure shift method. In Fig. 4 we show the result obtained from BEST-TROSY measurements for the disordered p53TAD^1−60^ (Dudas et al. 2020). To obtain high resolution, a relatively long acquisition time of 243 ms was chosen.

As can be seen in the highlighted 1D slices, a substantial enhancement in resolution is obtained. The corresponding correlation plot (Fig. 4c) reveals that the average signal width of 12.7 Hz for standard acquisition can be reduced to as low as 5.0 Hz using the ^13^C-BIRD-based pure shift sequence. Such a significant enhancement in resolution by a factor of ~ 2.5 is of major relevance for IDPs where overcrowded spectra are not only caused by low signal dispersion but frequently also by additional resonances originating from minor conformations.

From the collapse of multiplets one would expect that signal intensities are increased accordingly—the influence on sensitivity using pure shift, is, however, more complex. Both, the FHSQC and BEST-TROSY experiments make use of spin reservoirs that need to persist during the entire sequence and in turn allow high repetition rates. If such sensitivity-trimmed experiments are applied in combination with the ^13^C-BIRD-based pure shift acquisition, certain aspects with respect to sensitivity have to be reconsidered: (i) pure shift methods are committed to highest *resolution*—long acquisition times are hence mandatory, which dampens experimental repetition rates and should also be considered when using CPD (in HSQC) to avoid hardware damage; (ii) to a certain extent magnetization recovery of amide protons from longitudinal relaxation is hindered by repetitive pulsing during pure shift acquisition; (iii) an accelerated build-up of magnetization via NOE and spin diffusion (BEST approach) is not expected during the acquisition since the spin reservoir of surrounding protons is repeatedly flipped—an even number of chunks might partially retain the effect during the recovery delay, but particularly the recovery of reservoir polarization during acquisition does not take place; (iv) a faster magnetization build-up using the water spin reservoir is still possible, while, on the other hand, solvent exchange might induce line broadening which lowers the achievable resolution; (v) incomplete water suppression might enhance artificial noise originating from the chunked acquisition; (vi) the collapse of broad unresolved multiplets increases signal intensities to an extent that before-mentioned effects may well be compensated. Based on all these aspects, it is rather difficult to quantify the influence on sensitivity obtained for a general case when using the ^13^C-BIRD-based pure shift acquisition.

**Fig. 5:**
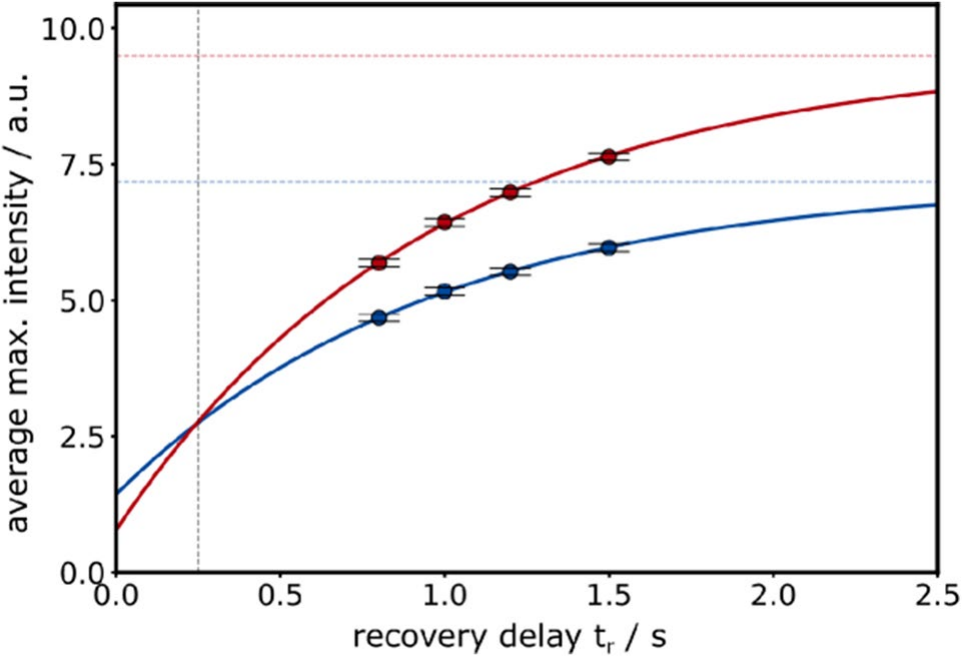
Average maximum intensities in a standard (blue) and a ^13^C-BIRD-based pure shift FHSQC (red) are fitted to a mono-exponential recovery function for ubiquitin. The acquisition time of 214 ms is split on 6 chunks with t_c_ = 17.8 ms using quadratic phase-shifted sine apodization

However, one can estimate the increase in sensitivity obtained by the pure shift FHSQC from Fig. 5 where standard and pure shift ubiquitin spectra are analyzed using four different recovery delays ${t}_{r}$ (ranging from 0.8 to 1.5 s not including acquisition times). In all spectra, maximum intensities of amide proton signals are extracted and average values are shown for standard (blue) and pure shift acquisition (red). The averaged data is fitted to mono-exponential recovery functions with $I ({t}_{r})={I}_{E}\cdot (1-{I}_{0}\cdot {e}^{-{R}_{1}{t}_{r}})$ where ${I}_{E}$ corresponds to the equilibrium magnetization, ${I}_{0}$ is the magnetization for ${t}_{r}=0$ and ${R}_{1}$ is the fitted average longitudinal relaxation rate. Note, repetitive pulsing interferes with magnetization buildup during real-time chunked acquisition and a lower value of ${I}_{0}$ is obtained for pure shift detection. From the fitting, it is hence possible to conclude that for very short recovery delays on average higher maximum intensities are found using standard acquisition (blue). Yet, already for recovery delays of t_r_ > 250 ms the gain in sensitivity due to collapsing multiplets outweighs the sensitivity of conventional acquisition and maximum intensities are on average increased by up to 32% using the ^13^C-BIRD-based pure shift detection. Note, total experimental times are increased using the decoupling scheme as shown in Table S1.

## Discussion

In the past decade various methods have been proposed for the real-time chunked acquisition of protein amide protons with which a significant enhancement in resolution can be achieved (Castañar et al. 2013; Ying et al. 2014; Kiraly et al. 2015). While in uniformly ^15^N-labeled proteins it is sufficient to suppress homonuclear ^1^H^N^,^1^H-couplings using band-selective or ^15^N-BIRD-based pure shift detection, numerous heteronuclear ^1^H^N^,^13^C-couplings cause significant line broadening if also ^13^C-spin labelling is applied. The straightforward use of ^13^C-CPD will lead to a dramatic increase in applied rf-energy during acquisition and appears exaggerated in isotropic liquids, where heteronuclear long-range couplings are almost two orders of magnitude smaller than typical ^1^*J*_CH_ couplings. It is hence adequate to include carbon decoupling in the real-time pure shift detection and high resolution can be obtained practically without hardware-limitation. Including the suppression of heteronuclear long-range couplings in the ^13^C-BIRD-based detection, we find that half chunk lengths (t_c_) of 15–20 ms are suitable to avoid considerable coupling evolution while keeping the interruptive nature of chunked acquisitions at a minimum. Possible small chunking sidebands from residual *J*-evolution may be suppressed by frequency or phase modulation, which is appropriate e.g. for IDPs where minor conformations induce additional sets of resonances with reduced intensities (Mauhart et al. 2015; Moutzouri et al. 2017).

It is further worth mentioning that transverse relaxation during chunking breaks (t_b_) introduces an artificial decay and the achievable line width is increased by a factor of $(2{t}_{c}+{t}_{b})/ (2{t}_{c})$ (Ying et al. 2014). For this reason, shorter breaks t_b_ are advantageous and the artificial decay induced by the ^13^C-BIRD-based acquisition (t_b_ ≈ 9.2 ms) is less pronounced than with the ^15^N-BIRD filter (t_b_ ≈ 13.2 ms). The shortest interruption is, however, found using optimized band-selective pulses (t_b_ ≈ 3–5 ms) and highest resolution would be obtained if it were not for the long-range heteronuclear couplings. For relaxation-limited samples hence, a subtle approach combining BASHD/HOBS with broadband ^13^C-inversions simultaneously applied to band-selective pulses might lead to the best compromise, which we, however, did not implement on our available, clearly more multiplet-broadened test samples. It is, however, crucial to note that the band-selective approach will fail for exchanging amide protons, since solvent suppression would require the saturation of the water resonance, thereby also removing corresponding amide signals.

## Concluding remarks

A novel selective element based on ^13^C-BIRD^r,X^ inversion for the real-time pure shift acquisition of amide protons in uniformly ^13^C,^15^N-labeled proteins has been introduced. An advantage of the method is, that numerous long-range ^1^H^N^,^13^C-couplings are suppressed without applying rf-intensive ^13^C-CPD, resulting in only a negligible effect on the duty cycle so that long acquisition times for highest resolution are still achievable. We present the application of this pure shift acquisition scheme incorporated in FHSQC and BEST-TROSY measurements for small folded proteins and IDPs. Experimental data reveal that on average the spectral resolution is increased by a factor of ~ 2 for the folded ubiquitin and ~ 2.5 for the IDP p53TAD^1-60^, respectively, while the average signal intensity in our example spectra was increased by approximately 30%. As saturation of water is not needed for solvent suppression we showed that the proposed pure shift sequence is perfectly suitable for amide proton detection of proteins. The obtained well-resolved 2D spectra are of great help in automated peak picking, and promotes a more straightforward assignment of overlapping peaks. The incorporation of the ^13^C-BIRD^r,X^ acquisition scheme into 3D and higher dimensional experiments should be straight-forward. It is especially suitable for ^13^C,^15^N-isotope-labeled IDPs where low natural linewidths allow a remarkable advantage from collapsing multiplets while solvent exchange needs to be considered for amide protons.

## Supplementary Information

Below is the link to the electronic supplementary material.

Supplementary file1 (DOCX 230 KB)

## Data Availability

The pulse sequences, raw and processed data will be available upon request.
