# Supplementary material for: Pure shift amide detection in conventional and TROSY-type experiments of 13C,15N-labeled proteins
Source: J Biomol NMR. 2022 Nov 18;76(5-6):213–21. doi: 10.1007/s10858-022-00406-z (PMC9712348; doi:10.1007/s10858-022-00406-z)
Supplement: Supplementary file 1 — Supplementary file1 (DOCX 230 KB) [file 10858_2022_406_MOESM1_ESM.docx]

**Supporting Information:**

**Pure Shift Amide Detection in Conventional and TROSY-type Experiments of ^13^C,^15^N-labeled Proteins**

Jens D. Haller, Andrea Bodor, Burkhard Luy

Contents

[Supporting Figures S1 and S2 2](#_Toc119503931)

[Supporting Table S1 3](#_Toc119503932)

[Setup of the pure shift FHSQC 4](#_Toc119503933)

[Setup of the pure shift BEST-TROSY 4](#_Toc119503934)

[Optimization of water suppression (gpz8) 5](#_Toc119503935)

[Pulse Sequence in Bruker format (FHSQC-type) 6](#_Toc119503936)

[Pulse Sequence in Bruker format (BEST-TROSY-type) 12](#_Toc119503937)

# Supporting Figures S1 and S2

**
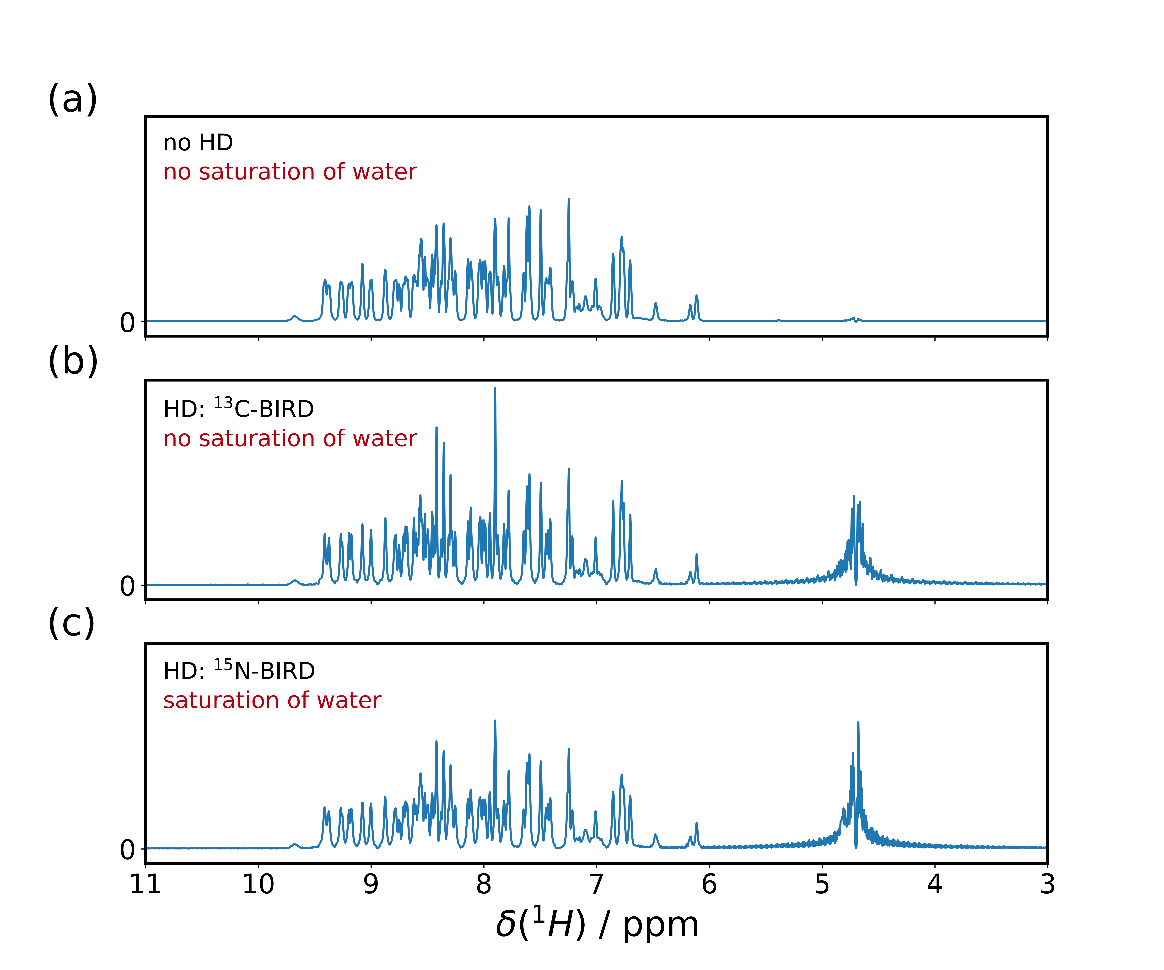
Figure S1.** 1D maximum projections are shown for the FHSQC with standard acquisition (a), using ^13^C-BIRD-based (b) and ^15^N-BIRD-based pure shift acquisition (c). Best water suppression is obtained for standard acquisition (a), while both pure shift methods achieve a similar level in solvent suppression. Note, in contrary to the ^15^N-BIRD-based acquisition, water does not need to be saturated when using ^13^C-BIRD.


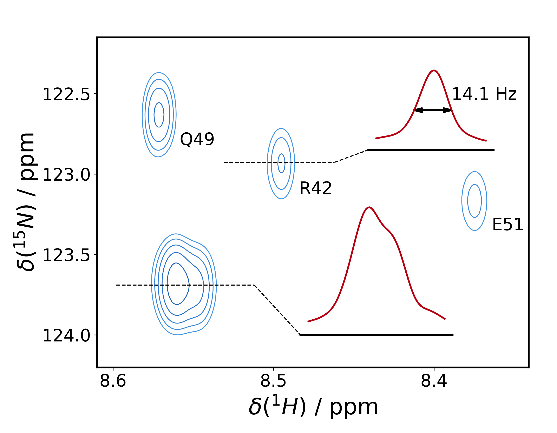

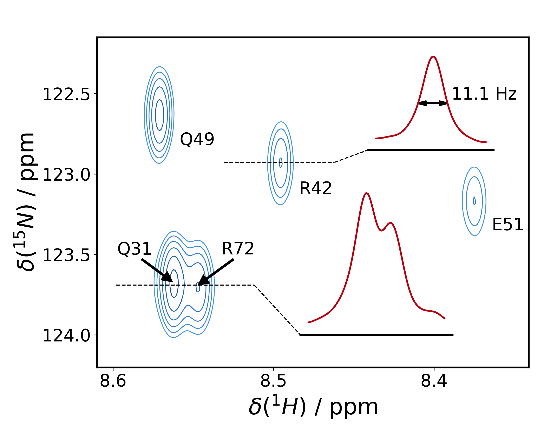

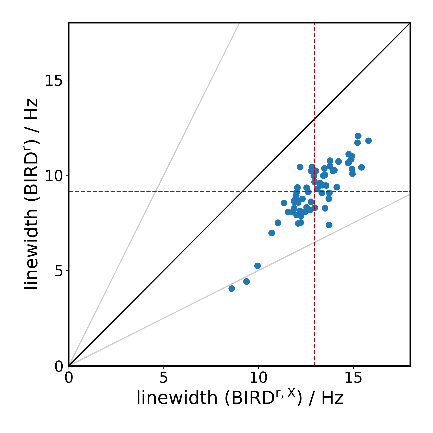
(a) (b) (c)

**Figure S2.** Spectra of ubiquitin acquired at 600 MHz using FHSQC with pure shift acquisition using a ^13^C-BIRD^r^ (a) and a ^13^C-BIRD^r,X^ filter (b). While long-range ^13^C-couplings are suppressed for (b) this is not the case in (a) and resolution is notably lower. In the direct dimension 2048 complex points were acquired in 214 ms with t_c_= 17.8 ms and n = 6 while in the indirect dimension 64 complex points were acquired in 30 ms with a magnetization recovery delay of t_r_= 1.0 s. Linewidths using ^13^C-BIRD^r^ and ^13^C-BIRD^r,X^ are compared in a correlation plot (c) and average linewidths (red dashed lines) of 12.9 Hz and 9.1 Hz are found, respectively. Note, the ^13^C-BIRD^r^ is slightly longer than the ^13^C-BIRD^r,X^ filter (with t_b_ = 9.46 ms instead of t_b_ = 9.14 ms), which, however, has only a negligible effect on the linewidth (<1%). A quadratic phase-shifted sine (a+b) and no apodization (c) was used for processing.

# Supporting Table S1

Typical parameters for the experimental setup with and without the proposed decoupling scheme.

| **FHSQC** | standard pulse sequence | with decoupling scheme |
| --- | --- | --- |
| spectral width (ppm) | 16 x 35 | 16 x 35 |
| time domain points (complex) | 2048 x 256 | 2048 x 256 |
| number of scans | 8 | 8 |
| acquisition time (ms) | 213.8 | 213.8 |
| recovery delay (s) | 0.8 | 0.8 |
| chunk numbers (l0) | 6 | 6 |
| total experimental time | 1h 15min 5s | 1h 24min 37s |

# Setup of the pure shift FHSQC

1. rpar: FHSQCF3GPPH

2. getprosol (and calibrate pulses)

3. Set pulse sequence (fhsqc_bbhd_13C) and gpnam8 (SMSQ10.100)
 A warning might pop up because chunk length tc (d63) and loop counter n (L0) are not yet set.

4. Use low power decoupling “garp4.p62” for longer acquisition times.

5. Set parameters:

cnst2: 140 Hz ^1^*J*_Hα,Cα_

gpz8: -3.0 % Must be further optimized for water suppression! → see next page.

gpz9: 0.3 % Gradient to prevent radiation damping
 NS: 8

DS: 32

l1: 4-12 Number of chunks → it will determine t_c_ (d63)

# Setup of the pure shift BEST-TROSY

1. rpar: B_TROSYETF3GPSI

2. getprosol (and calibrate pulses)

3. Set pulse sequence (b_trosy_bbhd_13C) and gpnam8 (SMSQ10.100)
 A warning might pop up because chunk length tc (d63) and loop counter n (L0) are not yet set.

4. Increase acquisition time (200ms or more) by increasing TD.

(→ Recall, it is a TROSY, so no composite pulse decoupling required.)

5. Set parameters:

cnst2: 140 Hz ^1^*J*_Hα,Cα_

D1: 0.2 s or more

gpz8: -3.0 % Must be further optimized for water suppression! → see next page.

gpz9: 0.3 % Gradient to prevent radiation damping
 NS: 4

DS: 32

l1: 4-12 Number of chunks → it will determine t_c_ (d63)

6. cnst26 gives the offset frequency for carbon inversion pulse. Check that Cαs and COs are covered.

7. Carbon bound protons need to be inverted for decoupling. Set L0 to even-numbered value and residual magnetization

of ^13^C-bound protons may still be retained for BEST approach.

# Optimization of water suppression (gpz8)

For optimal water suppression, we recommend the use of a shigemi tube.

The following instructions are intended for the use on Bruker spectrometers.

1. Copy the pure-shift experiment and make it a 1D version.

(Change parmode in “eda” → there is a button at the top that says “1, 2, ...” → click and choose “1D”)

2. In order to have best reproducibility, keep basic parameters as in 2D and only change:

NS: 1

DS: 4 or 8

Decrease receiver gain

3. Run a parameter optimization for gpz8. We recommend using “popt”, with parameters somewhat like:


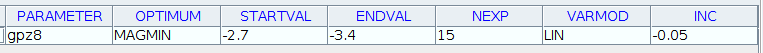


4. Choose gradient strength gpz8 with the least artefacts. Results should look similar to the figures below.

Note, for the optimization it is also possible to choose only the H_N_-area with command “dpl1” as shown in b).

1. b)


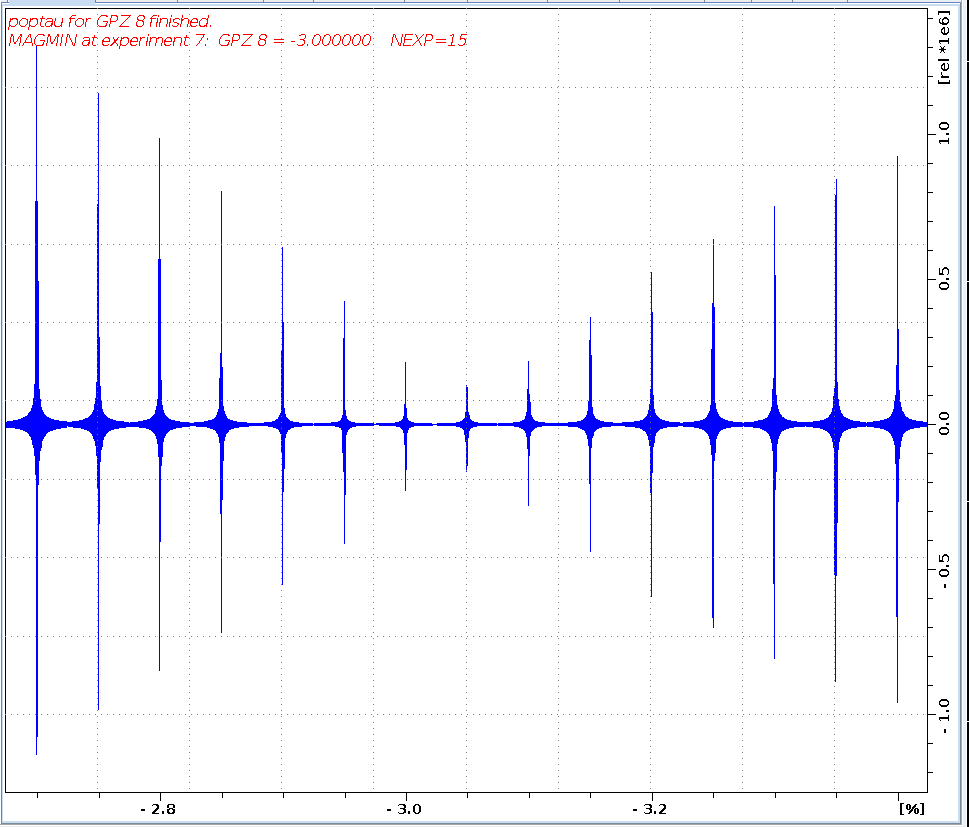

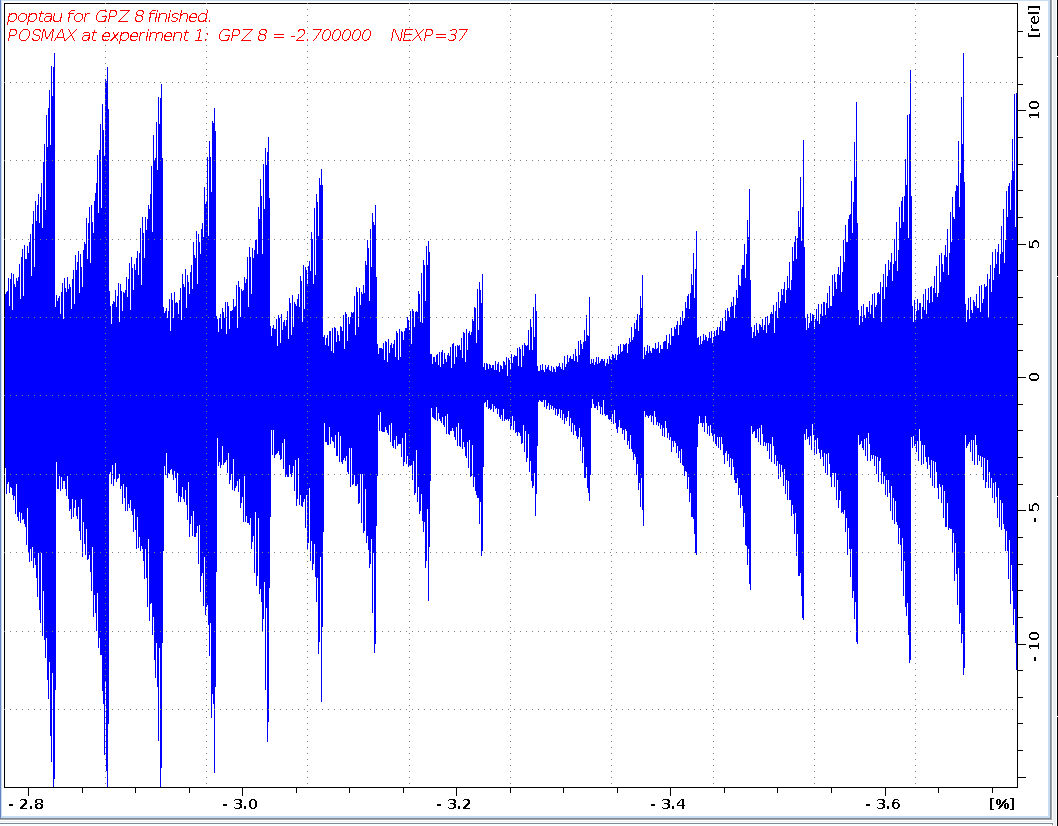


5. Transfer value of gpz8 to 2D and again determine receiver gain with updated value of gpz8.

6. A second parameter optimization of p1 using “popt” can further improve water suppression if pulses were not calibrated carefully beforehand. Setup can look something like:


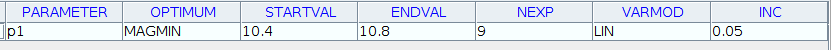


7. Avoid using linear prediction in the indirect dimension if water artifacts reach into the H_N_ area.

# Pulse Sequence in Bruker format (FHSQC-type)

#include <Avance.incl>

#include <Grad.incl>

#include <Delay.incl>

#include <De.incl>

"p2=p1*2"

"p22=p21*2"

"d11=30m"

"d12=20u"

"d13=4u"

"d21=1s/(cnst4*2)"

"d26=1s/(cnst4*4)"

"p29=300u"

"d0=3u"

"in0=inf1/2"

"DELTA=d19-p22/2"

"DELTA1=d26-p16-d16-p27*3-d19*5-p1*2/PI"

"DELTA2=d26-p16-d16-p27*2-p0-d19*5-de-8u"

"DELTA3=d0+larger(p2,p14)/2"

"DELTA4=p21*2/PI"

"DELTA5=d21-larger(p2,p22)/2"

"TAU=d26-p16-d16-4u"

;------homodecoupled acquisition

"d62=aq/l0"

"d63=d62/2"

"l1=l0-1"

"d22=1/(2*cnst2)"

"DELTA6=d22-larger(p2,p14)/2"

"d17=d16+p29+10u-8u"

;------homodecoupled acquisition

"acqt0=0"

baseopt_echo

dwellmode explicit

1 ze

d11 pl16:f3

2 d11 do:f3

4u BLKGRAD

d1

4u pl1:f1 pl3:f3

50u UNBLKGRAD

(p1 ph1)

4u

p16:gp1

d16

TAU

(center (p2 ph1) (p22 ph6):f3 )

TAU

4u

p16:gp1

d16

(p1 ph2)

4u

p16:gp2

d16

(p21 ph3):f3

DELTA3

(p22 ph3):f3

DELTA4

d0

(center (p2 ph5) (p14:sp3 ph1):f2 )

d0

DELTA4

(p22 ph4):f3

DELTA3

(p21 ph4):f3

4u

p16:gp2

d16

(p1 ph7)

DELTA1

p16:gp3

d16 pl18:f1

p27*0.231 ph2

d19*2

p27*0.692 ph2

d19*2

p27*1.462 ph2

DELTA

(p22 ph1):f3

DELTA

p27*1.462 ph8

d19*2

p27*0.692 ph8

d19*2

p0*0.231 ph8

4u

p16:gp3

d16

DELTA2 pl16:f3

4u cpd3:f3

;------homodecoupled acquisition

ACQ_START(ph30,ph31)

0.1u REC_UNBLK

0.05u DWL_CLK_ON

d63

0.05u DWL_CLK_OFF

0.1u REC_BLK

10u do:f3

p29:gp8

d16 rpp14 pl1:f1

;BIRD rX

(p1 ph11+ph14):f1

DELTA6

(center (p2 ph11+ph14):f1 (p14:sp3 ph11+ph14):f2 )

DELTA6

(p1 ph13+ph14):f1

4u gron9

d17

4u groff

4u gron9*-1

d17

4u groff

(p2 ph13+ph14):f1

p29:gp8*-1

d16

10u cpd3:f3

;loop

4 0.1u REC_UNBLK

0.05u DWL_CLK_ON

d62

0.05u DWL_CLK_OFF

0.1u REC_BLK

10u do:f3

p29:gp8

d16 ipp14

;BIRD rX

(p1 ph11+ph14):f1

DELTA6

(center (p2 ph11+ph14):f1 (p14:sp3 ph11+ph14):f2 )

DELTA6

(p1 ph13+ph14):f1

4u gron9

d17

4u groff

4u gron9*-1

d17

4u groff

(p2 ph13+ph14):f1

p29:gp8*-1

d16

10u cpd3:f3

lo to 4 times l1

0.1u REC_UNBLK

0.05u DWL_CLK_ON

d62*2

0.05u DWL_CLK_OFF

0.1u REC_BLK

rcyc=2

d11 do:f3 mc #0 to 2

F1PH(calph(ph3, +90) & calph(ph6, +90), caldel(d0, +in0))

exit

ph1=0

ph2=1

ph3=0 2

ph4=0 0 0 0 2 2 2 2

ph5=0 0 2 2

ph6=0

ph7=2

ph8=3

; bbhd

ph11=1 1 3 3

ph13=3 3 1 1

ph14=0 2 2 0 2 0 0 2

ph29=0

ph30=0

ph31=0 2 0 2 2 0 2 0

;pl1 : f1 channel - power level for pulse (default)

;pl3 : f3 channel - power level for pulse (default)

;pl16: f3 channel - power level for CPD/BB decoupling

;pl18: f1 channel - power level for 3-9-19-pulse (watergate)

;pl32: f1 channel - power level for low power presaturation

;sp3: f2 channel - shaped pulse 180 degree (adiabatic)

;spnam3: Crp60,0.5,20.1 (Crp80,0.5,20.1)

;p0 : f1 channel - 90 degree pulse at pl18

; use for fine adjustment

;p1 : f1 channel - 90 degree high power pulse

;p2 : f1 channel - 180 degree high power pulse

;p14: f2 channel - 180 degree shaped pulse for inversion (adiabatic)

;p16: homospoil/gradient pulse

;p21: f3 channel - 90 degree high power pulse

;p22: f3 channel - 180 degree high power pulse

;p27: f1 channel - 90 degree pulse at pl18

;p29: gradient pulse 3 [300 usec]

;d0 : incremented delay (2D) [3 usec]

;d1 : relaxation delay; 1-5 * T1

;d11: delay for disk I/O [30 msec]

;d12: delay for power switching [20 usec]

;d13: short delay [4 usec]

;d16: delay for homospoil/gradient recovery

;d19: delay for binomial water suppression

; d19 = (1/(2*d)), d = distance of next null (in Hz)

;d21 : 1/(2J(YH))

;d26 : 1/(4J(YH))

;d62: length of block between decoupling pulses : = aq/l0 [< 20-25 msec]

;d63: = d62/2

;cnst4: = J(YH)

;l0 : number of blocks during acquisition time

; adjust to get d62 as required

;inf1: 1/SW(X) = 2 * DW(X)

;in0: 1/(2 * SW(X)) = DW(X)

;nd0: 2

;ns: 8 * n

;ds: 16

;td1: number of experiments

;FnMODE: States-TPPI (or TPPI)

;cpd3: decoupling according to sequence defined by cpdprg3: garp4.p62

;pcpd3: f3 channel - 90 degree pulse for decoupling sequence

;cpdprg3: garp4.p62

gpz1: 50%

;gpz2: 80%

;gpz3: 30%

;gpz4: 3%

;gpz5: 5%

;use gradient files:

;gpnam1: SMSQ10.100

;gpnam2: SMSQ10.100

;gpnam3: SMSQ10.100

;gpnam4: SMSQ10.50

;gpnam5: SMSQ10.50

;preprocessor-flags-start

;LABEL_CN: for C-13 and N-15 labeled samples start experiment with

; option -DLABEL_CN (eda: ZGOPTNS)

;preprocessor-flags-end

;set pl32 to 0W when presaturation is not required

; use pl1 + 75 to 80dB to reduce radiation damping

# Pulse Sequence in Bruker format (BEST-TROSY-type)

#include <Avance.incl>

#include <Grad.incl>

#include <Delay.incl>

#include <De.incl>

define list<gradient> EA3 = { 1.0000 0.8750 }

define list<gradient> EA5 = { 0.6667 1.0000 }

define list<gradient> EA7 = { 1.0000 0.6595 }

"p22=p21*2"

"d11=30m"

"d12=20u"

"d25=2.7m"

"d26=2.7m"

"p29=250"

; Bruker standard values, don't change them

"cnst52=1.426"

"cnst53=1.0"

"cnst54=8.3"

"cnst55=5.0"

# ifdef CALC_SP

"p42=(bwfac26/(cnst55*cnst52*bf1))*1000000"

"spw26=plw1/((p42*90.0)/(p1*totrot26))*((p42*90.0)/(p1*totrot26))*(integfac26*integfac26)"

"spoal26=0.5"

"p43=(bwfac28/(cnst55*cnst53*bf1))*1000000"

"spw28=plw1/((p43*90.0)/(p1*totrot28))*((p43*90.0)/(p1*totrot28))*(integfac28*integfac28)"

"spw29=plw1/((p43*90.0)/(p1*totrot29))*((p43*90.0)/(p1*totrot29))*(integfac29*integfac29)"

"spoal28=1"

"spoal29=0"

# endif /*CALC_SP*/

"d0=3u"

"in0=inf1/2"

"DELTA1=d26-p19-d16-larger(p22,p42)/2"

"DELTA6=d25-p29-d16-larger(p22,p42)/2-p43*cnst43"

"DELTA7=d26-p16-d16-larger(p22,p42)/2"

"DELTA8=de+4u"

"DELTA=d0*2+p8+p21*4/PI"

"spoff13=bf2*(cnst26/1000000)-o2"

"spoff26=bf1*(cnst54/1000000)-o1"

"spoff28=bf1*(cnst54/1000000)-o1"

"spoff29=bf1*(cnst54/1000000)-o1"

"acqt0=0"

baseopt_echo

;------homodecoupled acquisition

"d62=aq/l0"

"d63=d62/2"

# ifdef BILEV

"d52=d62/4"

define list<delay> Dlist = { d63 d63 d63 d63 d52 d52 d52 d52 }

# else

define list<delay> Dlist = { d63 d63 d63 d63 }

# endif /*BILEV*/

"l1=l0-2"

"p2=2*p1"

"DELTA5=1/(2*cnst2)"

"d17=d16+p29-8u"

dwellmode explicit

;------homodecoupled acquisition

1 d11 ze

2 d11

3 d12

(p22 ph1):f3

20u BLKGRAD

d1

20u pl0:f1

50u UNBLKGRAD

(p43:sp28 ph3)

p19:gp1

d16

DELTA1

(center (p42:sp26 ph2) (p22 ph1):f3 )

DELTA1

p19:gp1

d16

(p43:sp29 ph2):f1

p16:gp2

d16

(p21 ph5):f3

d0

(p8:sp13 ph1):f2

d0

(p22 ph1):f3

DELTA

p16:gp3*EA3

d16

(p43:sp29 ph6)

p29:gp4

d16

DELTA6

(center (p42:sp26 ph2) (p22 ph2):f3 )

DELTA6

p29:gp4

d16

(p43:sp28 ph1)

p16:gp5*EA5

d16

DELTA8

(p21 ph1):f3

p16:gp6

d16

DELTA7

(center (p42:sp26 ph2) (p22 ph2):f3 )

DELTA7

p16:gp6

d16

(p21 ph7:r):f3

p16:gp7*EA7

d16

4u

;------homodecoupled acquisition

ACQ_START(ph30,ph31)

0.1u REC_UNBLK

0.05u DWL_CLK_ON

Dlist

0.05u DWL_CLK_OFF

0.1u REC_BLK

p29:gp8

d16 rpp14 pl1:f1

;BIRD rX

(p1 ph11+ph14):f1

DELTA5

(center (p2 ph11+ph14):f1 (p8:sp13 ph11+ph14):f2 )

DELTA5

(p1 ph13+ph14):f1

4u gron9

d17

4u groff

4u gron9*-1

d17

4u groff

(p2 ph13+ph14):f1

p29:gp8*-1

d16

; second acquisition

0.1u REC_UNBLK

0.05u DWL_CLK_ON

Dlist^

d63

0.05u DWL_CLK_OFF

0.1u REC_BLK

p29:gp8

d16 ipp14

;BIRD rX

(p1 ph11+ph14):f1

DELTA5

(center (p2 ph11+ph14):f1 (p8:sp13 ph11+ph14):f2 )

DELTA5

(p1 ph13+ph14):f1

4u gron9

d17

4u groff

4u gron9*-1

d17

4u groff

(p2 ph13+ph14):f1

p29:gp8*-1

d16

;loop

4 0.1u REC_UNBLK

0.05u DWL_CLK_ON

d62

0.05u DWL_CLK_OFF

0.1u REC_BLK

p29:gp8

d16 ipp14

;BIRD rX

(p1 ph11+ph14):f1

DELTA5

(center (p2 ph11+ph14):f1 (p8:sp13 ph11+ph14):f2 )

DELTA5

(p1 ph13+ph14):f1

4u gron9

d17

4u groff

4u gron9*-1

d17

4u groff

(p2 ph13+ph14):f1

p29:gp8*-1

d16

lo to 4 times l1

0.1u REC_UNBLK

0.05u DWL_CLK_ON

d62

d63

0.05u DWL_CLK_OFF

0.1u REC_BLK

rcyc=2

Dlist.res

d11 mc #0 to 2

F1EA(calgrad(EA3) & calgrad(EA5) & calgrad(EA7) & calph(ph6, +180) & calph(ph7, +180), caldel(d0, +in0) & calph(ph5, +180) & calph(ph31, +180))

4u BLKGRAD

exit

ph1=0

ph2=1

ph3=2

ph4=3

ph5=0 2

ph6=1

ph7=1

ph11=1 1 3 3

ph13=3 3 1 1

ph14=0 2 2 0 2 0 0 2

ph29=0

ph30=0

ph31=0 2

;pl1 : f1 channel - power level for pulse (default)

;pl3 : f3 channel - power level for pulse (default)

;sp13: f2 channel - shaped pulse 180 degree (Ca and C=O, adiabatic)

;sp26: f1 channel - shaped pulse 180 degree (Reburp.1000)

;sp28: f1 channel - shaped pulse 90 degree (Eburp2.1000)

;sp29: f1 channel - shaped pulse 90 degree (Eburp2tr.1000)

; for time reversed pulse

;p30 : f2 channel - 180 degree shaped pulse for inversion (BIP)

;p16: homospoil/gradient pulse [1 msec]

;p19: gradient pulse 2 [500 usec]

;p21: f3 channel - 90 degree high power pulse

;p22: f3 channel - 180 degree high power pulse

;p29: gradient pulse 3 [250 usec]

;p42: f1 channel - 180 degree shaped pulse for refocussing

; Reburp.1000 (1.4ms at 600.13 MHz)

;p43: f1 channel - 90 degree shaped pulse for excitation

; Eburp2.1000/Eburp2tr.1000 (1.7ms at 600.13 MHz)

;d0 : incremented delay (F1) [3 usec]

;d1 : relaxation delay; 1-5 * T1

;d11: delay for disk I/O [30 msec]

;d12: delay for power switching [20 usec]

;d16: delay for homospoil/gradient recovery

;d25: 1/(4J'(NH) [2.7 msec]

;d26: 1/(4J(NH)

;d62: chunk length

;d63: half a chunk length

;cnst2: 1J(Ca,Ha) coupling (140 Hz) [2.7 msec]

;cnst26: Call chemical shift (offset, in ppm) [101 ppm]

;cnst43: compensation of chemical shift evolution during p43

; Eburp2.1000: 0.69

;cnst52: scaling factor for p42 to compensate for transition region

; Reburp.1000: 1.426

;cnst53: scaling factor for p43 to compensate for transition region

; Eburp2.1000: 1.000

;cnst54: H(N) chemical shift (offset, in ppm)

;cnst55: H(N) bandwidth (in ppm)

;inf1: 1/SW(N) = 2 * DW(N)

;in0: 1/(2 * SW(N)) = DW(N)

;nd0: 2

;l0: number of chunks (normally 6 or 8)

;ns: 4 * n

;ds: 32

;td1: number of experiments

;FnMODE: echo-antiecho

;for z-only gradients:

;gpz1: 2%

;gpz2: 21%

;gpz3: -80%

;gpz4: 5%

;gpz5: 30%

;gpz6: 45%

;gpz7: 30.13%

;gpz8: -3.5% (change and see for best water suppression in 1Ds)

;gpz9: 0.5%

;use gradient files:

;gpnam1: SMSQ10.100

;gpnam2: SMSQ10.100

;gpnam3: SMSQ10.100

;gpnam4: SMSQ10.32

;gpnam5: SMSQ10.100

;gpnam6: SMSQ10.100

;gpnam7: SMSQ10.100

;gpnam8: SMSQ10.32

;preprocessor-flags-start

;CALC_SP: for calculation of all bandselective Proton pulses based on cnst54 and cnst55

; option -DCALC_SP (eda: ZGOPTNS) ;preprocessor-flags-end

;Processing

;PHC0(F1): 45.0
